# A Fully Integrated Memristive Chaotic Circuit Based on Memristor Emulator with Voltage-Controlled Oscillator

**DOI:** 10.3390/mi16030246

**Published:** 2025-02-21

**Authors:** Zhikui Duan, Jiahui Chen, Shaobo He, Xinmei Yu, Qiang Wang, Xin Zhang, Peng Xiong

**Affiliations:** 1 School of Electronic Information Engineering, Foshan University, Foshan 528225, China; duanzhikui@outlook.com (Z.D.); labxmyu@fosu.edu.cn (X.Y.); 2112203014@stu.fosu.edu.cn (Q.W.); 2112203018@stu.fosu.edu.cn (X.Z.); 2112203015@stu.fosu.edu.cn (P.X.); 2 School of Automation and Electronic Information, Xiangtan University, Xiangtan 411105, China

**Keywords:** memristor emulator, voltage-controlled oscillator, integrated circuit, chaotic circuit

## Abstract

This paper introduces a fully integrated memristive chaotic circuit, which is based on a voltage-controlled oscillator (VCO). The circuit employs a fully integrated architecture that offers reduced power consumption and a smaller footprint compared to the use of discrete components. Specifically, the VCO is utilized to generate the oscillatory signal, whereas the memristor emulator circuit serves as the nonlinear element. The memristor emulator circuit is constructed using a single operational transconductance amplifier (OTA), two transistors, and a grounded capacitor. This straightforward design contributes to diminished power usage within the chip’s area. The VCO incorporates a dual delay unit and implements current compensation to enhance the oscillation frequency and to broaden the VCO’s tunable range. Fabricated using the SMIC 180 nm CMOS process, this chaotic circuit occupies a mere 0.0072 mm^2^ of chip area, demonstrating a design that is both efficient and compact. Simulation outcomes indicate that the proposed memristor emulator is capable of operating at a maximum frequency of 300 MHz. The memristive chaotic circuit is able to produce a chaotic oscillatory signal with an operational frequency ranging from 158 MHz to 286 MHz, powered by a supply of 0.9 V, and with a peak power consumption of 3.5553 mW. The Lyapunov exponent of the time series within the resultant chaotic signal spans from 0.2572 to 0.4341.

## 1. Introduction

In 1971, Chua identified the absence of symmetry in the interrelation of circuit variables and introduced an element with the ability to delineate the correlation between magnetic flux and electric charge, known as the memristor [1]. Subsequently, in 1976, he delivered a comprehensive elucidation of its structural principles and functional attributes [2]. Owing to the nonexistence of tangible memristors, research pertaining to the memristor within the scientific and engineering disciplines remained constrained. It was not until 2008 that researchers at HP Labs devised a mathematical model for $\mathrm{Pt}/{\mathrm{TiO}}_{2}/\mathrm{Pt}$ devices within the conceptual framework of a memristor system, thereby inciting a proliferation of fervent research endeavors among a multitude of researchers [3]. Considering their capacity to retain information in the absence of a power supply, memristors have been the subject of extensive research for potential applications in nonlinear circuits [4,5], logic circuits [6,7,8], artificial neural networks [9,10,11], and nonvolatile memory [12,13,14].

Currently, memristors are becoming an important part of research in chaos science due to their unique nonlinear properties. Guseinov et al. [15] proposed a second-order hybrid memory system with capacitive and resistive components, in which hyper-periodic, intermittent, and chaotic behaviors may occur under periodic voltages. Ostrovskii et al. [16] introduced a dynamic threshold voltage function and a chaos generator based on the MMS model, and applied the proposed chaotic memristor model to develop an FPGA-based hardware memristor emulator. At the same time, adding a memristor to chaotic circuits has become a hot research topic. Guo et al. [17] proposed a modified Chua’s circuit in which a physical memristor ${\mathrm{Sr}}_{0.95}{\mathrm{Ba}}_{0.05}{\mathrm{TiO}}_{3}$ is used instead of the Chua’s diode in the Chua’s circuit. Laskaridis et al. [18] proposed a chaotic circuit based on a physical memristor that can behave as a static nonlinear resistor. Minati et al. [19] implemented a fully autonomous chaotic oscillatory circuit based on a physical memristor and a processed tungsten/carbon-based SDC memristor, and verified the bifurcation and multistable behavior in conjunction with variational equations. Meanwhile, discrete component-based memristive chaotic circuits have certain advantages in terms of applicability and portability, and much literature has been reported. Kengne et al. [20] designed a memristor-based chaotic oscillator, which is derived from the Shinriki circuit by substituting the nonlinear positive conductance with a first-order memristive diode bridge. Wu et al. [21] proposed a new memristor, and a new simple chaotic circuit was designed using the proposed memristor and other circuit components, which generates a chaotic attractor using only linear negative resistors, capacitors, inductors, and a memristor. Parnab Das et al. [22] present a novel four-dimensional chaotic system based on physical amnesia (TaOx), and a novel and efficient colour image encryption algorithm is developed based on this system. Ma et al. [23] present a simple novel chaotic oscillatory circuit that can generate 19 different types of attractors by building them in parallel with a memristor, memcapacitor, and inductor. Ostrovskii et al. [24] proposed a bio-inspired neuron model based on a threshold selector and tunnel diode, with a bidirectional threshold switch selector device, as an example of a non-volatile memristor that mimics the dynamic behavior of neurons. The above-mentioned memristive chaotic circuits are designed by replacing the nonlinear item in the original system with a memristor. Furthermore, the study of memristive Chua’s circuits has also aroused much interest. Escudero et al. [25] proposed a physical implementation of a tunable memristor Chua’s circuit, based on a non-volatile memristor device, by programming the non-volatile memory device into different states to select between different oscillation modes. Bao et al. [26] designed a fifth-order two-memristor Chua’s circuit and hyperchaos was observed.

Integrated circuits provide advantages such as reduced power consumption and compact designs. The aforementioned chaotic circuits, which predominantly utilize commercial discrete devices, not only suffer from high power consumption, high costs, and extensive footprint, but also have instability issues. The integration of chaotic circuits can significantly mitigate these shortcomings. Consequently, over the past few decades, numerous researchers have conducted rigorous research on this subject. Nguyen et al. [27] presented a low-power hyperchaos-based true random number generator, which is based on 130 nm CMOS technology. Valencia [28] proposed a chaotic system based on approximate fractional derivatives and implemented the circuit using 180nm CMOS integrated circuit technology. Eduardo et al. [29] proposed a CMOS implementation of a Lorentzian system circuit, which is implemented by operational transconductance amplifiers as well as multipliers, and used this Lorentzian system to implement a CMOS secure communication system. Joshi et al. [30] proposed a simple CMOS-based chaotic oscillator with a circuit structure consisting of capacitors as well as MOS tubes and an external comparator circuit as a nonlinear term. Although the mentioned integrated chaotic circuits have achieved integration, they still exhibit notable flaws in power consumption and footprint, with pronounced deficiencies in oscillation frequency. Meanwhile, most proposed integrated chaotic circuits have exhibited low oscillation frequencies. Consequently, this paper introduces a fully integrated memristive chaotic circuit based on a ring oscillator and a memristor emulator. The circuit comprises a ring oscillator, a memristor emulator circuit, two ground capacitors, and a resistor. By introducing a ring oscillator to generate high-frequency oscillation signals, the performance of the chaotic circuit is improved. Meanwhile, we designed the integrated circuit of the memristor; thus, the integration level of the chaotic circuit can be guaranteed.

The subsequent sections of this paper are organized as follows. Section 2 delineates the architecture of the proposed memristive chaotic circuit and the memristor emulator. Section 3 describes the architecture of the proposed voltage-controlled ring oscillator circuit, along with the comprehensive topology and mathematical model of the memristive chaotic circuit. The simulation outcomes and performance validation of the proposed memristive chaotic circuit are presented in Section 4. Finally, the conclusion is succinctly summarized in Section 5.

## 2. Design of a Memristive Chaotic Circuit

### 2.1. The Simplest Memristive Chaotic Circuit

**Figure 1:**
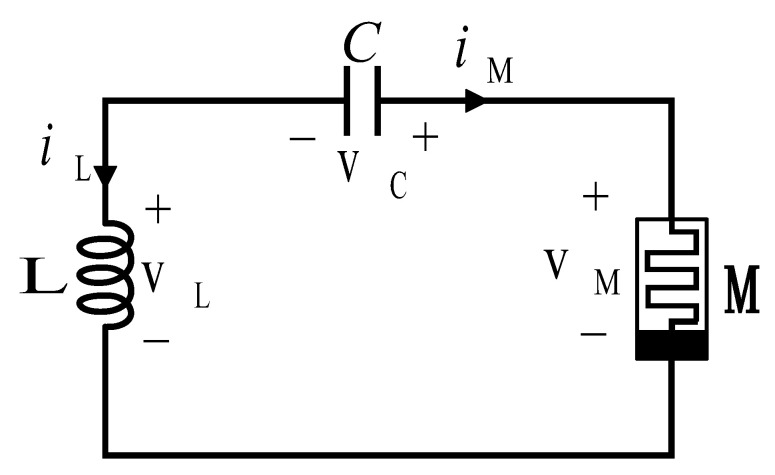
The simplest memristive chaotic circuit.

Muthuswamy et al. [31] designed a memristive chaotic circuit, as depicted in Figure 1, which they refer to as the simplest memristive chaotic circuit. As shown in Figure 1, the circuit comprises a capacitor denoted as *C*, an inductor labeled as *L*, and a current-controlled memristor designated as *M*.

In the architecture of the memristive chaotic circuit under examination, capacitors and inductors serve as dual-purpose energy repositories, simultaneously establishing the requisite oscillatory conditions for chaotic phenomena and preserving their energy storage functionalities. The memristor acts as a generator of nonlinear dynamics, thus completing the foundational triad that forms the essential configuration for the generation of chaos. The traditional approach utilizes a multi-component architecture that includes operational amplifiers, analog multipliers, and discrete passive elements. Nevertheless, this modular design introduces significant constraints regarding power efficiency and spatial compactness, essentially limiting its suitability for integration into contemporary system-on-chip architectures.

**Figure 2:**
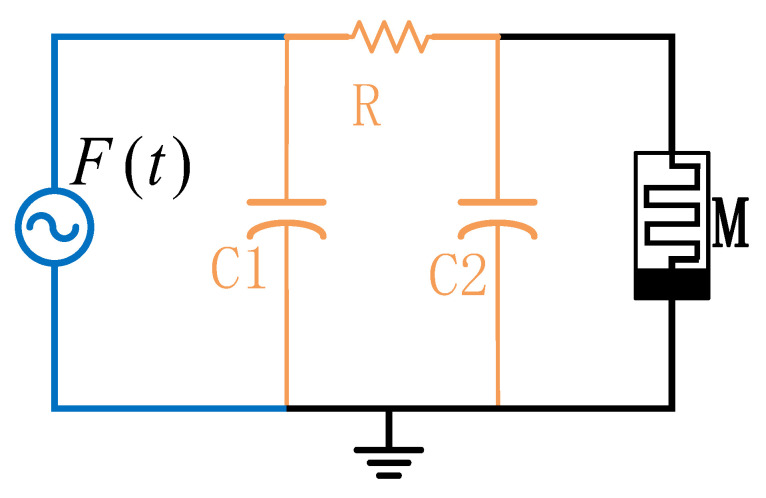
The proposed memristive chaotic circuit.

In order to mitigate these limitations, we propose a monolithic integrated memristive chaotic circuit, depicted in Figure 2. The suggested architecture amalgamates three principal components: (1) an active oscillation core responsible for generating foundational chaotic signals, (2) resistively coupled capacitive banks functioning as synchronized energy storage nodes, and (3) a CMOS-compatible memristor emulator performing essential nonlinear transformations. This architectural synthesis attains compactness while maintaining chaotic operational characteristics through the amalgamation of functions.

### 2.2. Design of the Memristor Emulator

#### 2.2.1. The Memristor Emulator Circuit

The memristor emulator circuit replicates the characteristics of the memristor through correlation within the circuitry, and its voltage–current characteristics exhibit nonlinear properties. The resistance of the memristor can be modulated by regulating the intensity and duration of the electrical pulse signals that pass through it.

**Figure 3:**
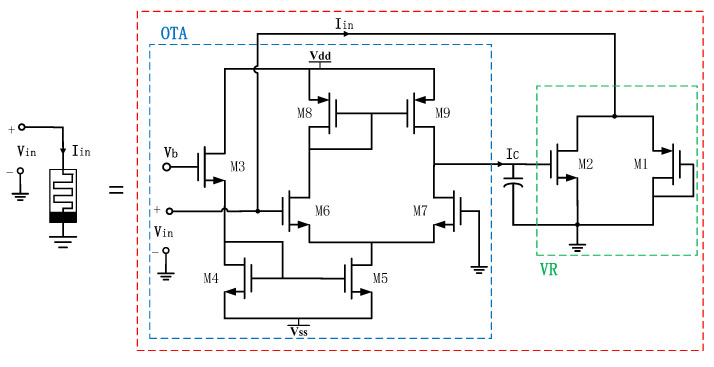
Circuit topology of memristor emulator.

The memristor emulators delineated in this article consist of an operational transconductance amplifier (OTA), a variable resistor (VR), and ground capacitors, which utilize a DC voltage supply of $V_{dd}=+0.9$ V and $V_{ss}=-0.9$ V. The circuit topology is depicted in the accompanying Figure 3, wherein the blue box signifies the OTA circuit, and the green box signifies the VR circuit.

The variable resistor, composed of the two source–drain connected PMOS and NMOS transistors discussed in this article, has a resistance value that is contingent upon two control voltages. Specifically, the gate voltage of M2 represents the capacitor voltage value, denoted as $V_{C}$, while the voltage at the source–drain junction of M1 and M2 constitutes the control voltage, denoted as $V_{cont}$. Within this configuration, $M_{1}$ constitutes the structure of the MOS diode. When $M_{1}$ operates within the saturated region and $M_{2}$ within the linear region, the input current can be articulated as

(1) $$I_{1}\;=\;\frac{k_{1}}{2}{\left(V_{cont}-V_{TH_{1}}\right)}^{2}$$

(2) $$I_{2}\;=\;k_{2}\left[\left(V_{c}-V_{TH_{2}}\right)V_{cont}-\frac{V_{cont}^{2}}{2}\right]$$

where *k* is the process parameter of the MOS transistor and can be expressed as

(3) $$k_{1}\;=\;\mu_{p}C_{OX}{\left(\frac{W}{L}\right)}_{1}$$

Here, $\mu_{p}$, $\frac{W}{L}$, and $C_{OX}$ are the mobility of charge carrier, the aspect ratio, and the oxide capacitance per unit area of MOS, respectively. According to the drain current formula of the two MOS transistors, it can be concluded that $k_{1}$ and $k_{2}$ are the same. They have good matching characteristics, and the total current flowing through the VR can be expressed as

(4) $$I_{in}=k\left[\left(V_{C}-\left|V_{TH_{1}}\right|-V_{TH_{2}}\right)V_{cont}+{\left|V_{TH_{1}}\right|}^{2}/2\right]$$

The output current of the OTA for an input is expressed as

(5) $$I_{out}=G_{m}\left(V_{N}-V_{P}\right)$$

$G_{m}$ in the above equation is the transconductance value of the OTA. The analysis results in the expression of Gm as

(6) $$G_{m}=\frac{k_{6,7}}{\sqrt{2}}\left(V_{b}-V_{SS}-2V_{TH_{3}}\right)$$

The current $I_{C}$ flowing into the grounding capacitor can be expressed as

(7) $$I_{C}\;=\;I_{out}\;=\;G_{m}V_{in}$$

Bias voltage $V_{C}$ can be expressed as

(8) $$V_{C}=\frac{1}{C}\int I_{C}\left(t\right)dt\;=\;\frac{G_{m1}}{C}\int V_{in}\left(t\right)dt\;=\;\frac{G_{m}\phi_{in}}{C}$$

As $\left|V_{TH1}\right|$ $\leq V_{cont}$ ≤ $V_{C}$ − $V_{TH2}$, Equation (4) can be expressed as

(9) $$I_{in}\;=\;I\;=\;k\left[\left(\frac{G_{m}\phi_{in}}{C}-\left|V_{TH_{1}}\right|-V_{TH_{2}}\right)V_{in}+{\left|V_{TH_{1}}\right|}^{2}/2\right]$$

So, the model for the proposed memristor emulator circuit can be expressed as

(10) $$W\left(\phi_{in}\right)\;=\;\frac{I_{in}}{V_{in}}\;=\;k\left[(\frac{G_{m}\phi_{in}}{C}-\left|V_{TH_{1}}\right|-V_{TH_{2}})\right]$$

#### 2.2.2. Simulation Results

To scrutinize the frequency response of the proposed emulator design, encompassing both time-invariant and time-variant components, a bipolar input signal $V_{in}$ = $V_{m}sin\omega t$ was applied. The computation of the input flux $\left(\phi_{in}\right)$ is executed in accordance with the following procedure:

(11) $$\phi_{in}\;=\;-\frac{A_{m}cos\omega t}{\omega }$$

Substituting the value of flux of Equation (11) in Equation (10), the memductance can be calculated as follows:

(12) $$W\left(\phi_{in}\right)\;=\;-k\left(\frac{G_{m}A_{m}cos\omega t}{\omega C}\right)-k\left(\left|V_{TH_{1}}\right|-V_{TH_{2}}\right)$$

This approach allows us to analyze the emulator’s behavior under different frequency conditions, providing valuable insights into its performance characteristics.

According to Equation (12), the first part is a time-varying quantity and the second part is a constant. This complies with the generalized memristor simulator equation.

**Figure 4:**
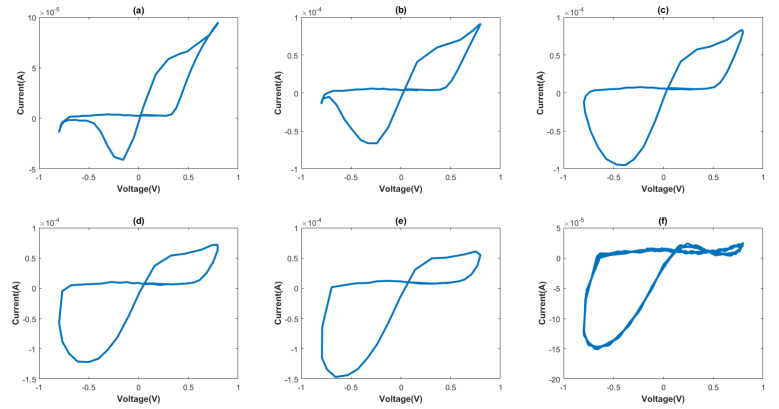
Pinch hysteresis curve for different frequencies, when frequency is (**a**) 100 MHz, (**b**) 150 MHz, (**c**) 200 MHz, (**d**) 250 MHz, (**e**) 300 MHz, and (**f**) 350 MHz.

In order to verify the characteristics of the proposed memristor, frequency characteristic and non-volatile analysis of the memristor are carried out. In the frequency characteristic analysis, we applied a sine signal with a frequency variation from 100 to 350 MHz to the circuit, with a fixed MOS capacitor dimension value of 5 × 5 μm; the resulting pinch hysteresis curves are shown in Figure 4.

Examination of the graph reveals that the proposed memristor circuit displays an “8”−shaped hysteresis loop, but distortion emerges with increasing signal frequency. However, within the frequency range of 100 MHz to 200 MHz, the memristor circuit demonstrates a superior hysteresis curve. Consequently, the operating frequency for this integrated chaotic circuit was chosen to fall within this frequency range.

**Figure 5:**
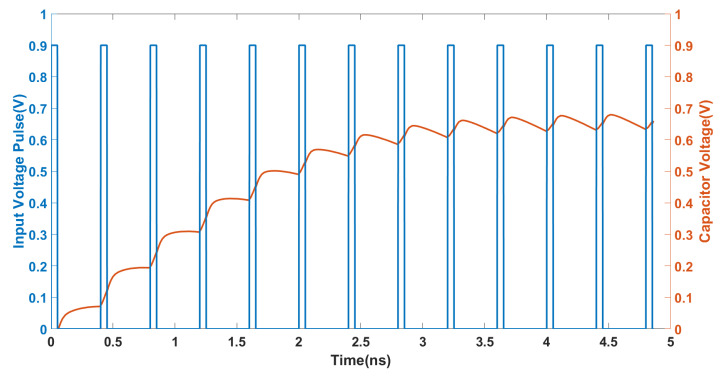
Non−volatility analysis for proposed memristor circuit.

The non-volatile properties are one of the most important properties of the memristor. In order to further verify the non-volatile properties of the memristor circuit, a series of voltage pulses as input and the corresponding changes in the memductance values were observed. Figure 5 shows the pulse of the applied input voltage along with the voltage of the capacitor. According to Equations (8) and (10), we can obtain the memductance values. Table 1 shows the CMOS dimension of the memristor simulator. This amnesia simulator has the advantages of a high operating frequency and low number of active components.

Finally, we give a comparison between the memristor emulator circuit proposed in this paper and the previously proposed memristor emulator circuits, and the results of the analysis are shown in Table 2. A comparison of the structure of the memristor emulator circuits in the table reveals that the memristor emulator proposed in this paper employs fewer circuit modules and passive components than other models. Furthermore, it is capable of achieving a higher operating frequency while using a straightforward OTA structure, which is more straightforward than the overall circuit.

## 3. Integrated Circuit Design and Its Analysis

### 3.1. The Voltage−Controlled Oscillator

#### 3.1.1. Implementation of the Voltage−Controlled Oscillator

In integrated circuits, signal generators are commonly embodied by oscillators. Ring oscillators offer numerous significant advantages over LC voltage-controlled oscillators (LC VCOs). They eliminate the need for inductive components, conserve chip real estate, reduce costs, provide a broad tuning range, and facilitate easy multiphase implementation. However, ring oscillators typically exhibit slightly inferior phase noise performance. Considering its benefits of low power consumption, a small footprint, and high integration, a ring oscillator is chosen as the voltage-controlled oscillator for implementation.

**Figure 6:**
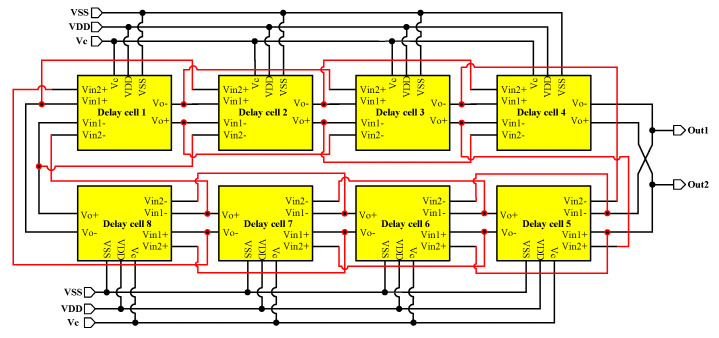
Implementation of voltage-controlled oscillators.

**Figure 7:**
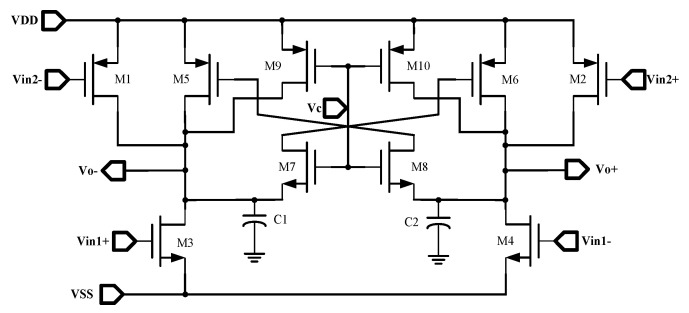
Circuit topology of double delay cells in voltage−controlled oscillators.

Figure 6 illustrates the circuit structure of the ring voltage-controlled oscillator, where the red line indicates the second loop of the delay unit. The VCO consists of eight basic delay units, and the circuit structure of the delay units is shown in Figure 7, and the transistor widths and lengths of the delay cells are shown in the Table 3. The delay unit uses a dual delay path, with the input path Vin1+ (Vin1−) connected to the output of the previous delay unit, and the input path Vin2+ (Vin2−) connected to the input path Vin1+ (Vin1−) of the previous level. The input path Vin2, because it is connected to the input Vin1 of the previous level, provides an advance phase to compensate for the slow turn−on of the pull−up pMOS transistor as a means of speeding up the pmos tubes and thus increasing the oscillation frequency of the VCO [40]. A pair of PMOS load transistors, M5 and M6, are incorporated into the delay cell to construct the latch. Furthermore, the cross-coupled NMOS pass transistors, M7 and M8, govern the maximum gate voltage of the PMOS load transistors (M5 and M6), and modulate the strength and operating frequency of the added latch. An additional pair of PMOS transistors, M9 and M10, are employed to enhance the frequency control range of the VCO, mitigating the limited frequency control range resulting from the reduced latch strength at lower control voltages.

The integration of these components not only optimizes the performance of the latch but also ensures a more stable and efficient operation across a wide range of frequencies. This design approach enhances the overall functionality and reliability of the VCO, making it suitable for applications that demand precise frequency control and stable operation.

The tuning gain represents the output frequency versus the input reference voltage, written as $K_{VCO}$, and expressed as

(13) $$K_{VCO}=\frac{f_{max}^{\prime }-f_{min}^{\prime }}{V_{max}-V_{min}},$$

**Figure 8:**
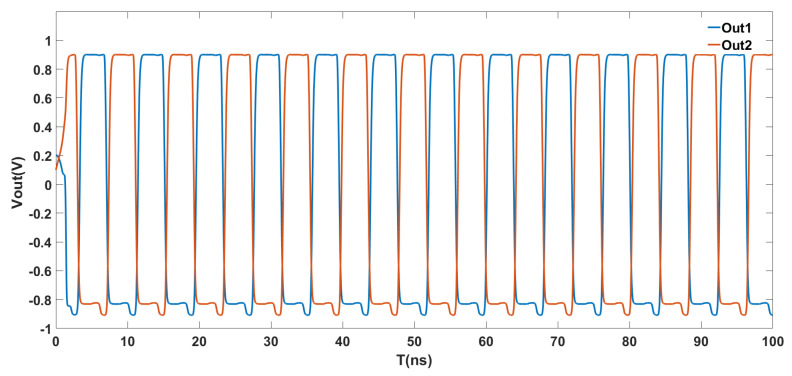
Out1−Out2 output curve of VCO.

When the $K_{VCO}$ of the VCO is large, the VCO is more sensitive to the control voltage $V_{C}$. Since the input voltage $V_{C}$ is not an ideal signal, the spuriousness of the input voltage is amplified, which affects the noise characteristics and thus the tuning range of the VCO. The output frequency of the control voltage $V_{C}$ is expressed as

(14) $$f_{out}^{\prime }=f_{0}+K_{VCO}V_{C}$$

where $f_{0}$ refers to the output frequency when $V_{c}=-0.9$ V, (Vmax−Vmin) is the maximum and minimum value of the control voltage $V_{c}$, and K is a constant. It can be seen from the equation that a large $K_{VCO}$ can be obtained and a small change in the voltage $V_{c}$ can be controlled to cause a large change in the output frequency. Figure 8 represents the two output waveforms of the VCO proposed in this paper, with a supply of ±0.9 V and a control voltage $V_{C}$ of −0.3 V. The frequency of the oscillating signal generated is 284 MHz.

#### 3.1.2. Process Corner Simulation

The parameters of MOSFETs can vary significantly across different wafers, posing challenges for circuit designers. To address this, process engineers are dedicated to ensuring that equipment performance remains within acceptable parameters. They achieve this by rigorously controlling the expected parameter variations, ensuring they remain within the maximum range that the chip’s performance envelope can withstand. This acceptable range of performance is commonly referred to as a “process corner” and is provided to designers to help them account for these variations in their circuit designs.

**Figure 9:**
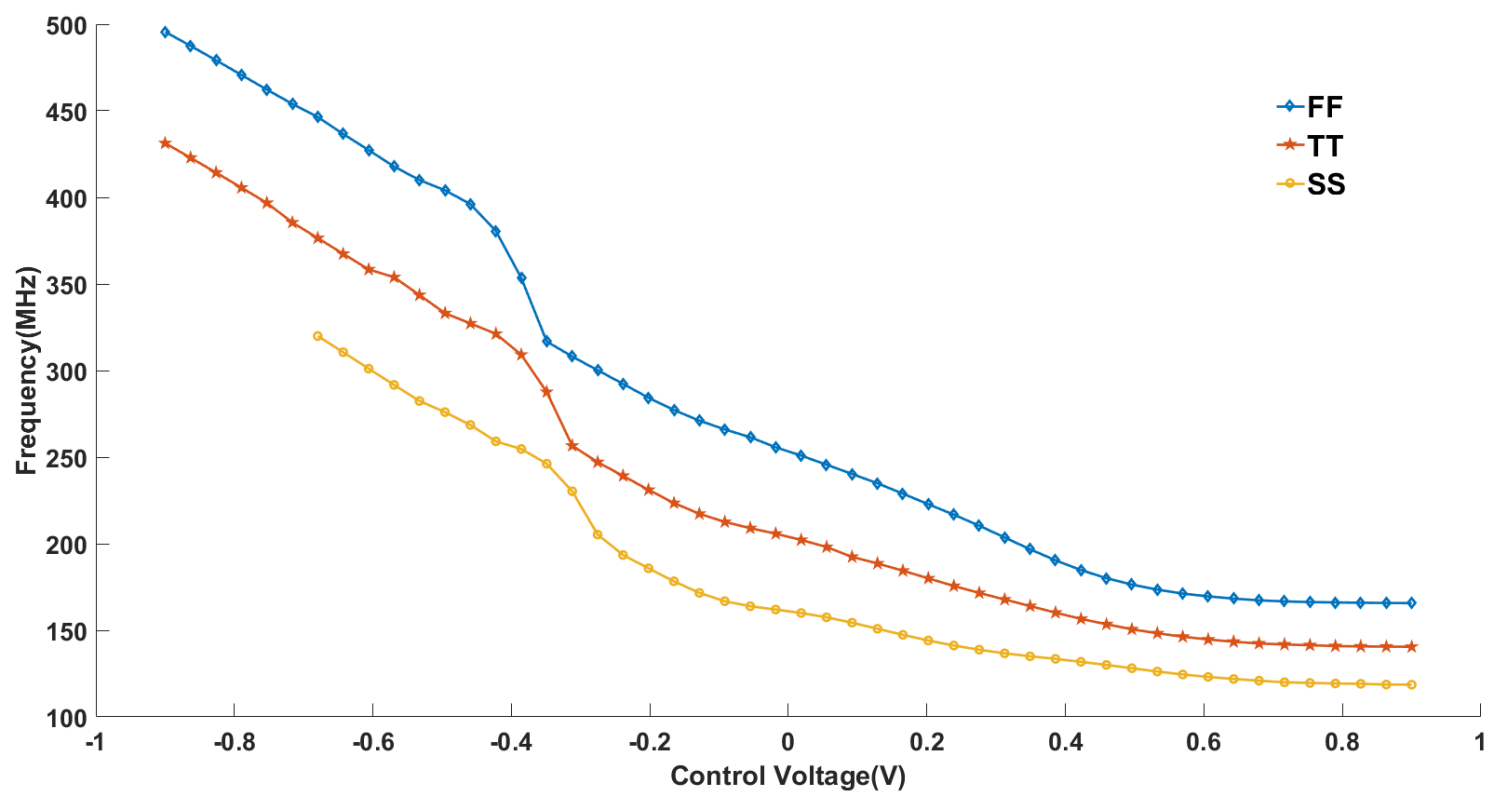
$K_{VCO}$ curves under different PVT processes.

As shown in Figure 9, we carried out process corner experiments for the variation in oscillator output frequency with control voltage; as shown in the figure, we carried out the experiments for Fast MOS, Typical MOS, and Slow MOS processes, respectively [41]. We found that the oscillator has differences in various process angles but the overall performance is very close.

### 3.2. *Design of the Proposed Memristive Chaotic Circuit Based on a VCO*

**Figure 10:**
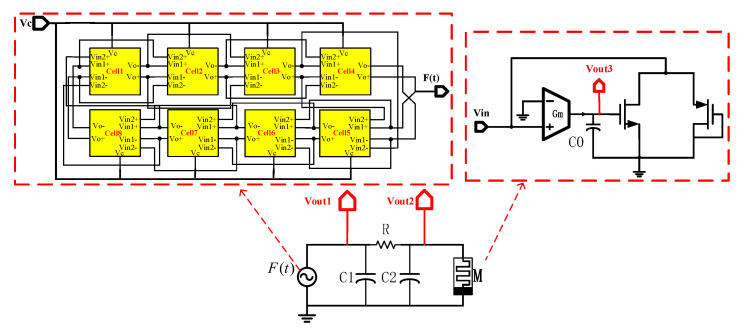
Proposed memristive chaotic circuit dased on VCO.

The structure of the proposed chaotic circuit based on a VCO is shown in Figure 10. The signal generator comprises eight connected delay cells on the left side of the circuit, with the nonlinear component being formed by a memristor circuit on the right side. Because a voltage-controlled oscillator is used instead of an external input signal, the chaotic system can be considered as an autonomous chaotic system. In order to meet the conditions of chaos in the autonomous chaotic system, an energy storage component capacitor is added to the oscillator.

Based on Kirchhoff’s law, we can derive the following equation of state for the circuit in Figure 10:

(15) $$\left\{\begin{array}{l} \frac{dV_{out2}}{dt}=\frac{V_{out1}-V_{out2}}{RC_{2}}-\frac{W\left(V_{out3}\right)V_{out2}}{C_{2}}\\ \frac{dV_{out1}}{dt}=\frac{V_{out2}-V_{out1}}{RC_{1}}+\frac{V_{out1}}{8R_{osc}C_{1}}\\ \frac{dV_{out3}}{dt}=\frac{G_{m}}{C_{0}}V_{out2} \end{array}\right.$$

where $V_{out1}$ is the voltage at the point where the oscillator and capacitor $C_{1}$ are connected in parallel, $V_{out2}$ is the voltage at the parallel connection of the nonlinear term and capacitor $C_{2}$, $R_{osc}$ is the output impedance of the oscillator, and $W(\phi_{in})$ denotes the memductance value of the memristor emulator, which can be expressed by the following equation:

(16) $$W\left(V_{out3}\right)\;=\;k\left[(V_{out_{3}}-\left|V_{TH_{1}}\right|-V_{TH_{2}})\right]$$

where $V_{out3}$ represents the capacitor voltage in the memristive circuit. In this formulation, the variables and components interact to determine the overall behavior of the circuit, highlighting the role of each element in the system.

The above equations can be used to derive dimensionless parametric equations that are applicable to chaotic systems:

(17) $$\left\{\begin{array}{l} \dot{x}=\alpha \left(y-x\right)-a*b\left(z-c\right)x\\ \dot{y}=\beta \left(x-y\right)+r*y\\ \dot{z}=\gamma *x \end{array}\right.$$

In the above dimensionless parametric equations, the parameters are determined by the device parameters in the circuit:

(18) $$\left\{\begin{array}{c} \alpha \;=\;\frac{1}{RC_{2}},\beta \;=\;\frac{1}{RC_{1}},\gamma \;=\;\frac{G_{m}}{C_{0}},b\;=\;k,\\ r\;=\;\frac{1}{8R_{osc}C_{1}},a\;=\;\frac{1}{C_{2}},c\;=\;\left|V_{TH_{1}}\right|+V_{TH_{2}} \end{array}\right.$$

where $C_{0}$ = 50 fF, Gm = 861.29 μS, *k* = 1.6308 mA/V^2^, *c* = 904.9 mV, $R_{osc}$ = 12 kΩ, *R* = 2 k∼7 kΩ, $C_{1}$ = 400 fF, and $C_{2}$ = 800 fF.

In this paper, an integrated voltage-controlled oscillator is used to generate oscillating signals in place of the sinusoidal signal generator in the non-autonomous chaotic circuit. The nonlinear term is used in an integrated memristor circuit, which mimics the nonlinear characteristics of the memristor. The integrated design of the overall circuit makes the proposed chaotic circuit advantageous in terms of power consumption, footprint, and stability.

## 4. Layout Design and Simulation Verifications

### 4.1. The Layout Design

**Figure 11:**
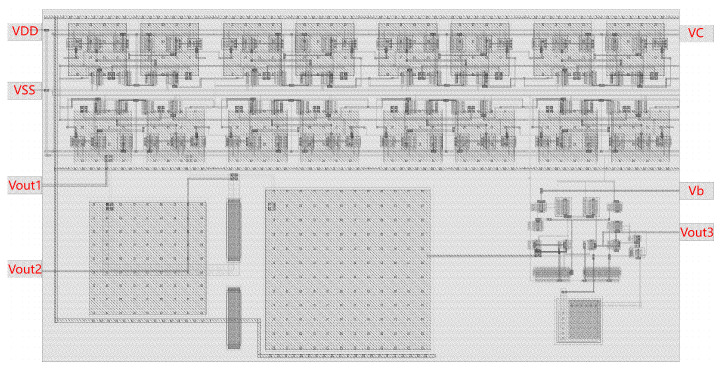
Proposed memristive chaotic circuit layout.

The layout of the proposed VCO−based memristive chaotic circuit in this paper is shown in Figure 11. The overall area occupied by the layout is 0.0072 mm^2^. In this work, a fully integrated VCO−based memristive chaotic circuit is realized using the SMIC 180 nm CMOS process technology.

### 4.2. Simulation Verifications

In this section, we discuss SPICE simulations performed on the proposed fully CMOS oscillator−based memristive chaotic circuit and present the results. The total power consumption of the integrated circuit comprises both static and dynamic components. The static power dissipation $P_{static}$, primarily attributed to device leakage currents, can be expressed as follows: $P_{static}=I_{leak}\times V_{DD}$, where $I_{leak}$ denotes the cumulative leakage current through all transistors.The dynamic power consumption $P_{dynamic}$, arising from switching activities, is determined by $P_{dynamic}=C_{load}\times V_{DD}^{2}\times f_{clock}$, where $C_{load}$ represents the effective load capacitance, and $f_{clock}$ is the operating frequency. Key parameters influencing power consumption include supply voltage, operating frequency, and nodal capacitance. SPICE simulations at ±0.9 V dual supply voltages yield a total power dissipation of 3.5553 mW.

To generate chaotic phenomena, adjustments are made to various circuit parameters, including capacitance, resistance, and the aspect ratio of the transistors. Determining the correct parameter settings has become crucial, as these are not only based on design constraints but also take into account the characteristics of the entire system. We individually adjusted the system’s operating frequency and device parameters to achieve various chaotic phenomena. Subsequently, we employed Rosenstein’s small-data method to calculate the Lyapunov exponent and utilized the 0–1 test to validate its chaotic effects.

#### 4.2.1. Chaotic Phenomena at Different Oscillation Frequencies

**Figure 12:**
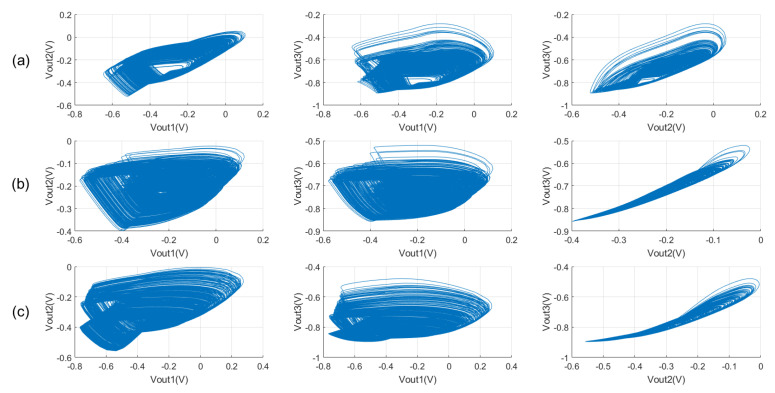
Chaotic phenomena with different oscillation frequencies at Tran = 1 ps, T = 5 µs, and $V_{b}$ = 0.3 V: (**a**) f = 158 MHz, R = 2 KΩ, $C_{0}$ = 50 fF, $C_{1}$ = 300 fF, $C_{2}$ = 500 fF, and Ly = 0.2572; (**b**) f = 211 MHz, R = 5 KΩ, $C_{0}$ = 50 fF, $C_{1}$ = 500 fF, $C_{2}$ = 1200 fF, and Ly = 0.4047; and (**c**) f = 284 MHz, R = 5.2 KΩ, $C_{0}$ = 50 fF, $C_{1}$ = 400 fF, $C_{2}$ = 800 fF, and Ly = 0.4341.

Figure 12 illustrates the phase diagram showing the relationship between outputs Vout1, Vout2, and Vout3. Each phase diagram corresponds to different device parameters and oscillation frequencies. In the simulation experiments, we vary the device parameters ($R,C_{0},C_{1},C_{2}$) and give three sets of chaotic phase diagrams and Lyapunov exponents Ly. In the first set of phase diagrams (a), the control voltage $V_{c}$ of the voltage-controlled oscillator is 0.4 V, the oscillation frequency is 158 M, and the device parameters are as follows: *R* = 2 KΩ, $C_{0}$ = 50 fF, $C_{1}$ = 300 fF, $C_{2}$ = 500 fF, and Ly = 0.2572. In the second set of phase diagrams (b), the control voltage $V_{c}$ of the voltage-controlled oscillator is −0.1 V, the oscillation frequency is 211 M, and the device parameters are as follows: *R* = 5 KΩ, $C_{0}$ = 50 fF, $C_{1}$ = 500 fF, $C_{2}$ = 1200 fF, and Ly = 0.4047. Finally, in the phase diagram of the third group (c), the control voltage $V_{c}$ of the voltage-controlled oscillator is −0.4 V, the oscillation frequency is 284 M, and the device parameters are as follows: *R* = 5.2 KΩ, $C_{0}$ = 50 fF, $C_{1}$ = 400 fF, $C_{2}$ = 800 fF, and Ly = 0.4341.

Examining these phase diagrams, we observe distinct chaotic behaviors at varying operating frequencies of the oscillator. The phase diagrams exhibit a certain complexity. Moreover, the Lyapunov exponent being greater than zero confirms that the system is in a chaotic state. Despite the chaotic behaviors observed at various operating frequencies, the corresponding Lyapunov exponents are not high. The reason is that simply adjusting the operating frequency and the parameters of the coupling device between the oscillator and the nonlinear term is not sufficient to continuously amplify the chaotic phenomenon. Therefore, to achieve the desired chaotic effect, adjustments must be made to the parameters of each circuit component and the voltage bias. By observing the phase diagram, we can infer that chaotic behavior may be present in the system, but these observations are not sufficient to fully assess the chaotic sophistication of the system.

#### 4.2.2. Effect of Different Device Parameters on Chaotic Phenomena

**Figure 13:**
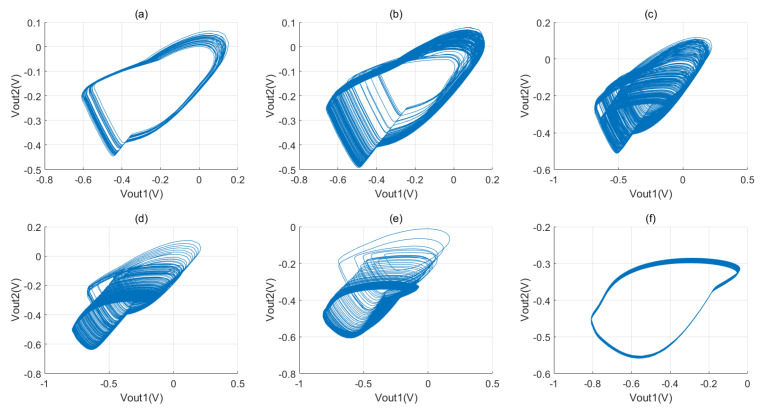
Chaotic phenomena with different values of *R* and operating frequency of 298 MHz: (**a**) R = 4 KΩ, Ly < 0; (**b**) R = 4.5 KΩ; (**c**) R = 4.8 KΩ; (**d**) R = 5.5 KΩ; (**e**) R = 7 KΩ; and (**f**) R = 10 KΩ, Ly < 0.

In Figure 13, we maintain the parameters of all the devices of the system except the resistance constant and systematically increase the resistance value to investigate the effect of the device parameters on the chaotic phenomena. From Figure 13a,b, the phase diagram becomes progressively more complex, with a resistance range of 4 K to 4.5 K. From Figure 13c,d, the phase diagram shows a notable complexity, with a resistance range of 4.8–5.5 K. The resistance range from Figure 13a,d results in the beginnings of chaos that gradually increase in intensity. From Figure 13e,f, with a resistance range of 7 K to 10 K, the phase diagram shows a diminishing chaotic behavior that stabilizes in Figure 13f, indicating that it is no longer in a chaotic state.

In summary, chaotic systems feature a specific range of variables that greatly influence chaotic phenomena. The complexity of a phase diagram does not necessarily indicate pronounced chaotic effects in a system. Identifying a range of variables alone does not lead to the best chaotic phenomenon. To attain the desired chaotic effect, one must employ mathematical tools, such as the Lyapunov exponent, to determine the precise parameters of the system.

#### 4.2.3. Validation of Chaos

In order to validate the chaotic effects of the proposed fully integrated memristive chaotic circuit, the Lyapunov exponent and 0−1 chaos tests were conducted. The Lyapunov exponent is a crucial quantitative measure of a system’s dynamic properties; this is a numerical feature that represents the mean exponential divergence of adjacent trajectories in phase space. In this study, the orbit tracking method is utilized to monitor orbits of the system, thereby enabling the determination of their evolutionary patterns and the extraction of Lyapunov exponents.

In addition, the 0−1 chaos test was performed to further confirm the chaotic behavior. This test involves analyzing the system’s response to initial conditions, as minute variations can lead to significantly different outcomes, a hallmark of chaotic systems. The outcomes of these two analytical procedures were found to be concordant, thus providing substantial evidence for the existence of chaos in the aforementioned circuit.

**Figure 14:**
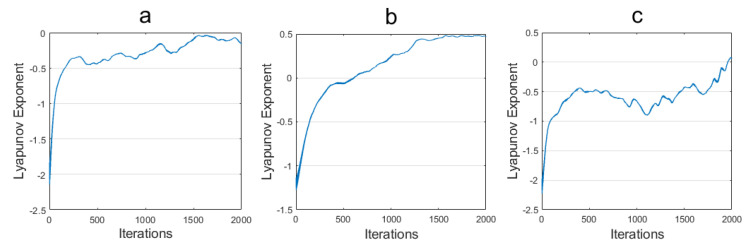
Corresponding Lyapunov exponents for (**a**) Figure 13a; (**b**) Figure 13d; and (**c**) Figure 13e.

The Lyapunov exponent in Figure 13a,d,e, with a data length of 2000, was extracted and analyzed. The results are presented in Figure 14, which shows that the Lyapunov exponent is consistently less than 0 and has not entered the chaotic state in part (a). In part (b), the Lyapunov exponent is observed to increase and subsequently stabilize at approximately 0.5, indicative of a chaotic state. In contrast, the Lyapunov exponent in part (c) is predominantly negative and exceeds 0 in the final segment.

The 0−1 test is a test algorithm for measuring the presence of chaotic time series in a time series. It was first proposed by Li and Yorke (1975) [42] in their study of periodic solutions in nonlinear dynamical systems. The algorithm is specifically defined by the following equation:

(19) $$\begin{array}{c} p_{c}\left(n\right)=\sum_{i=1}^{n}\phi \left(i\right)cos\left(\alpha \left(i\right)\right))\\ q_{c}\left(n\right)=\sum_{i=1}^{n}\phi \left(i\right)sin\left(\alpha \left(i\right)\right)) \end{array}$$

Furthermore, the displacement mean square error M(n) is utilized as a measurement tool, which is calculated in accordance with the following Equation (20). Consequently, the K-value equation is obtained.

(20) $$M\left(n\right)=\lim_{N\to +\infty }\frac{1}{N}\sum_{N}^{i=1}{\left[p\left(i+n\right)-p\left(i\right)\right]}^{2},n=1,2\ldots$$

(21) $$K\;=\;\lim_{n\to +\infty }\frac{\mathrm{lg}M(n)}{\mathrm{lg}n}$$

**Figure 15:**
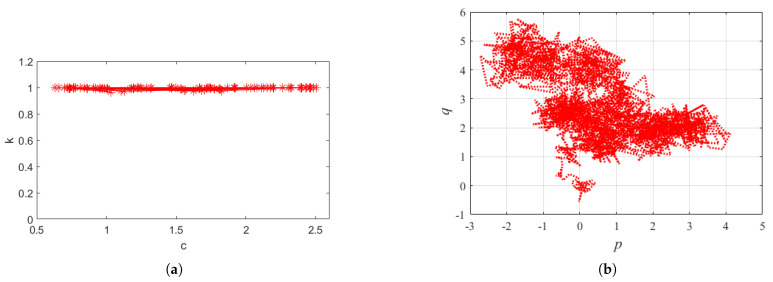
(**a**) The verification result graph of the 0−1 test algorithm. (**b**) The p−q planar graph of the 0–1 test algorithm.

We use the Matlab tool for simulation verification of the 0−1 test algorithm. The plots of *p* and *q* of the phase diagram of Figure 13c proposed in this paper are given in Figure 15b, and the dynamics is chaotic.

By calculating the value of K, the result is shown in Figure 15a. It can be seen that its value is close to 1, indicating that the system is indeed in a chaotic state.

#### 4.2.4. Performance Comparison

Table 4 shows the comparison of this work with other chaotic work. It is clear from the table that for the realization of chaotic circuits, more studies have chosen to build them using separated devices, which are not comparable to integrated chaotic circuits in all aspects of performance. Compared with other chaotic circuits implemented using CMOS integrated circuits, the work in this paper outperforms the other articles in terms of circuit power consumption, footprint, and output frequency. It is certain that integration, as well as high performance and low power consumption, are inevitable trends in the development of circuits.

## 5. Conclusions

To align with current industry developments, circuits must meet key requirements, including power consumption, stability, portability, and performance. Because there are many shortcomings in circuits composed of discrete components, the implementation of integrated circuits is a new trend. Therefore, this paper introduces a chaotic integrated circuit that utilizes memristive circuits and voltage-controlled oscillators, effectively reducing power consumption and footprint while enhancing stability and performance. The circuit is fabricated using SMIC 180 nm CMOS technology, occupies an area of 0.0072 mm^2^, consumes 3.5553 mW of power, and operates at a maximum frequency of 286 MHz. As a result, when the polyphase output signals and low-mode area are more of a concern, a VCO is preferable in conventional circuits. Meanwhile, with the continuous reduction in the CMOS feature size, the transistor size is smaller and faster, which can meet the trend of integrated circuit development and market demand. In the future, we will design chaotic chips for secure communication to face even greater challenges and opportunities.

## Figures and Tables

**Table 1:** Transistor dimensions for memristor emulator.

MOS Transistor	W/L	MOS Transistor	W/L
M1	2.6 µm/0.2 µm	M2	0.6 µm/0.2 µm
M3	1.2 µm/0.6 µm	M4	1.2 µm/0.6 µm
M5	6.0 µm/0.6 µm	M6	1.2 µm/0.2 µm
M7	1.2 µm/0.2 µm	M8	2.5 µm/0.5 µm
M9	2.5 µm/0.5 µm		

**Table 2:** Transistor width−to−length ratio in memristor emulator.

[Ref]	Active Component	Passive Components	Supply Voltage (V)	Operating Frequency
[32]	1CFTA	1C	±1.2	9 MHz
[33]	1VDCC, 1OTA	1C	±0.9	30 MHz
[34]	1CCII, 1OTA	1R, 1C	±1.2	26.3 MHz
[35]	1CDBA, 1OTA	1C	±0.9	1 MHz
[36]	1VDBA, 1OTA	1MOS-C	±0.9	5 MHz
[37]	1VDTA, 2MOS	-	±0.9	50 MHz
[38]	1VDCC, 1OTA	1C	±0.9	8 MHz
[39]	2MO-OTA	1C	±1.2	400 KHz
This Work	1OTA, 2MOS	2R,1C	±0.9	300 MHz

**Table 3:** Transistor dimensions for delay cell.

MOS Transistor	W/L	MOS Transistor	W/L
M1	1.0 µm/0.2 µm	M2	0.6 µm/0.2 µm
M3	1.2 µm/0.6 µm	M4	1.2 µm/0.6 µm
M5	6.0 µm/0.6 µm	M6	1.2 µm/0.2 µm
M7	1.2 µm/0.2 µm	M8	2.5 µm/0.5 µm
M9	2.5 µm/0.5 µm		

**Table 4:** Comparison of CMOS chaotic circuit proposed in this paper with other chaotic circuits.

[Ref]	Structure	Frequency (MHz)	Supply Voltage (V)	Power (mW)	Process (µm)	Chip Area (mm^2^)
[14]	Autonomous Continuous	45.12	1.2	0.677	0.13	-
[30]	Comparator	5	±0.9	29.6	0.18	-
[43]	Single-Delay VCO	0.011–0.036	1.8	2.0892	0.18	0.039
[44]	OTRA	7.8	±1.25	23	0.25	-
[45]	DVCCTA	0.0027	±9	23	0.25	-
[46]	Chaotic PWM	1.2	3.3	-	0.18	0.626
[47]	Bit Generator	-	1.8	1.32	0.18	0.037
This Work	memristor VCO	158–286	±0.9	3.5553	0.18	0.0072

## Data Availability

The original contributions presented in this study are included in the article. Further inquiries can be directed to the corresponding authors.
